# Response of the daily transpiration of a larch plantation to variation in potential evaporation, leaf area index and soil moisture

**DOI:** 10.1038/s41598-019-41186-1

**Published:** 2019-03-18

**Authors:** Yunni Wang, Gongxiang Cao, Yanhui Wang, Ashley A. Webb, Pengtao Yu, Xiaojiang Wang

**Affiliations:** 1 Inner Mongolia Academy of Forestry Sciences, Hohhot, 010010 China; 2 Institute of Forest Ecology, Environment and Protection, Chinese Academy of Forestry, Beijing, 100091 China; 3 NSW Department of Primary Industries, Tamworth Agricultural Institute, Calala, NSW 2340 Australia; 4 Present Address: WaterNSW, PO Box 1251, Tamworth, NSW 2340 Australia

## Abstract

Tree transpiration (T) is a major water budget component and varies widely due to the integrated effects of many environmental and vegetation factors. This study aimed to separate, quantify, and then integrate the effects of the main individual factors, to improve water use estimation and manage the hydrological impacts of forests. A field study was conducted at 3 plots of larch (*Larix principis-rupprechtii*) plantation in the semi-humid area of the Liupan Mountains, northwest China. The main influencing factors were the atmospheric evaporative demand expressed by potential evapotranspiration (PET), the soil water availability expressed by volumetric soil moisture (VSM) within the 0–100 cm layer, and the canopy transpiration capacity expressed by forest canopy leaf area index (LAI). The daily stand T was estimated through the up-scaling of sap-flow data from sampled trees. It displayed a high degree of scattering in response to PET, VSM and LAI, with an average of 0.76 mm·day^−1^ and range of 0.01–1.71 mm·day^−1^ in the growing season of 2014. Using upper boundary lines of measured data, the response tendency of T to each factor and corresponding function type were determined. The T increases firstly rapidly with rising PET, VSM and LAI, then gradually and tends to be stable when the threshold of PET (3.80 mm·day^−1^), VSM (0.28 m^3^·m^−3^) and LAI (3.7) is reached. The T response follows a quadratic equation for PET and saturated exponential function for VSM and LAI. These individual factor functions were coupled to form a general daily T model which was then fitted using measured data as: *T* = (0.793*PET* − 0.078*PET*^2^)·(1 − exp(−0.272*LAI*))·(1 − exp(−9.965*VSM*)). It can well explain the daily T variation of all 3 plots (R^2^ = 0.86–0.91), and thus can be used to predict the response of daily T of larch stands to changes in both environmental and canopy conditions.

## Introduction

In the dryland regions of China, vegetation restoration has been implemented for several decades through large-scale afforestation programs such as the “Three-North Forest Shelterbelt” Program since 1978, the “Natural Forest Protection” Program since 1998, and the “Grain for Green” Program since 1999, with the main purpose of protecting soil against serious erosion and sandstorms^1–4^. Further afforestation activities have been planned as a measure to strengthen the carbon sequestration function of forests (http://www.gov.cn/ldhd/2009-09/23/content_1423825.htm). However, as a result of ignoring the vegetation carrying capacity of limited water resources, the large-scale afforestation has induced several unexpected problems, such as soil desiccation^4–6^, “little-old-trees”^7^ and water yield reduction^8–10^. This threatens the forest stability, forest ecosystem services, and the regional water supply security and sustainable development. Therefore, the accurate estimation of water consumption of forests under changing environmental conditions is urgently required.

Tree transpiration (T) is an important physiological and hydrological process^11,12^ and the main cause of water loss from forest ecosystems in dryland regions^9,13^, thus directly affecting the forest stability and water yield from forestland and forested watersheds. The T varies widely due to the effects of numerous factors^13^, which can be simplified and divided into three aspects: the potential evapotranspiration of the atmosphere (PET)^14,15^, the water supply ability from root zone soil^13,15–17^ and the water conveying capacity of vegetation^18–20^.

Across a broad range of species and ecosystems, it was found that the T initially increases quickly and almost linearly with rising soil moisture to a threshold^15,16,21–25^, then gradually to approach its potential maximum. However, the threshold value has varied among different studies. The response of T to soil moisture can be described with logistic or quadratic relations, or segmented linear relations. Most such studies were designed for understanding the T response only to soil drying and were conducted in pots or chambers under controlled weather conditions. In the limited field studies, the data during cloudy, overcast, rainy days or those with extreme vapor pressure deficit (VPD) were often excluded, to derive a moderate relation between T and soil moisture^16,26–28^, so such results cannot fully reflect the T behavior in the field. In most cases, the forest T is controlled not only by soil moisture, except during the periods with high enough soil water deficit^16,23,29,30^, but also strongly by the wide variation of atmospheric evaporative demand and available energy^13,31,32^, which can be simply represented by the integrated index of PET^29,33^. In addition, the dynamic leaf amount in canopy is the most important active factor influencing the water conveying ability of forests, and inevitably leads to the T variation. The T increases nonlinearly with rising LAI, as confirmed in a controlled study^34^.

Most studies on forest T variation have considered only one or two of the influencing factors of soil moisture, meteorological factors and LAI. For example, Li *et al*.^33^ studied the T of a larch plantation in the middle of the growing season to analyze the effect of PET and relative extractable soil water (REW), but without consideration of LAI. The lack of field studies, which consider the coupled effects of all the main influencing factors, limits the understanding and accurate prediction of forest T under changing environmental conditions, and the integrated forest-water management in a multifunctional way.

Thus, to describe the variation of daily T of forest stands under changing PET, VSM and LAI, and to evaluate the effects of these factors, as well as to predict the T variation with a comprehensive model, this study was carried out in 3 plots of Prince Rupprecht’s larch (*Larix principis-rupprechtii*) plantation at the Liupan Mountains area located in the transitional zone between the semi-humid and semi-arid regions of northwest China.

## Results

### Variations of LAI, weather and soil moisture

**Figure 1:**
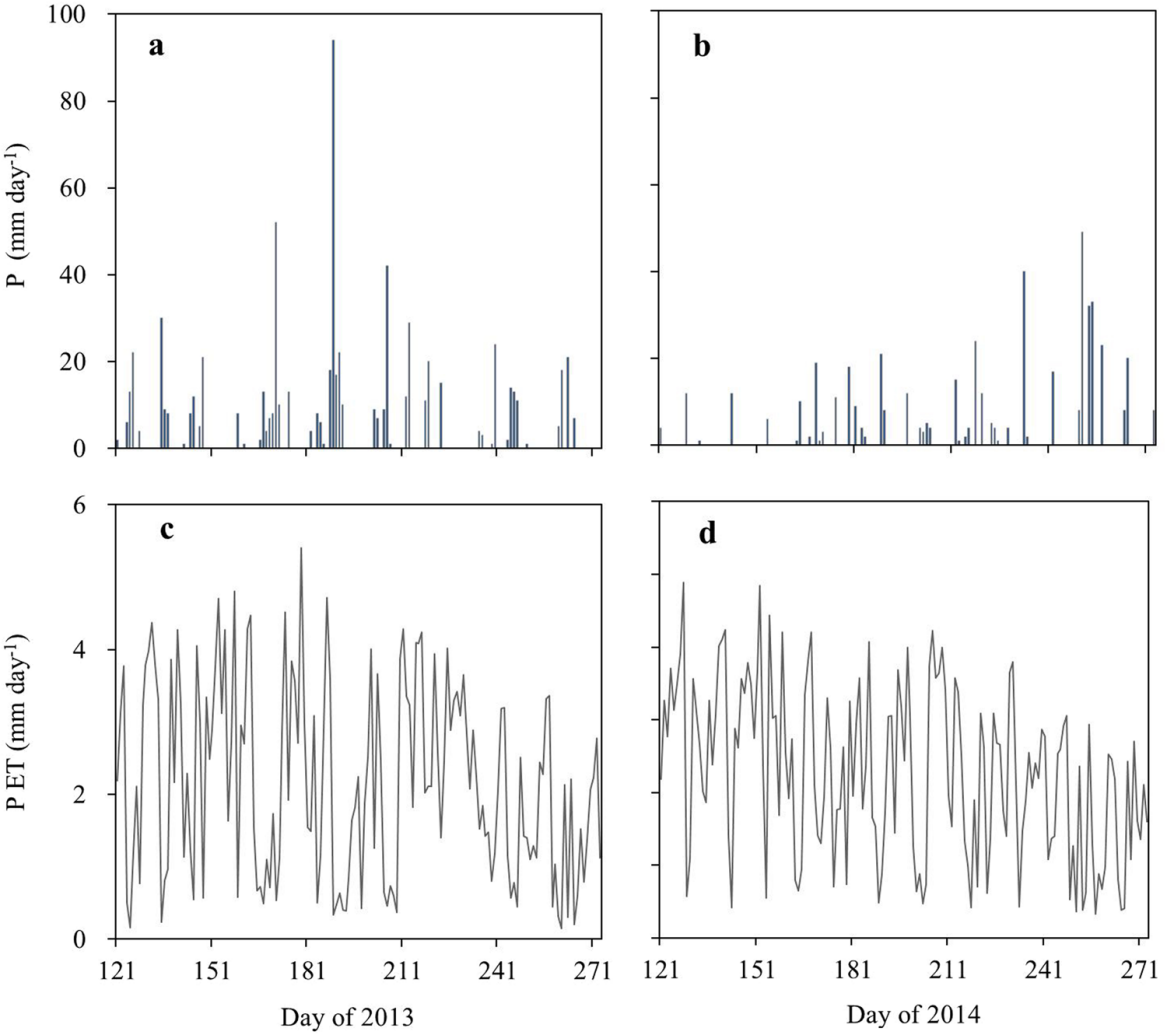
Variations of daily precipitation (**a** and **b**) and PET (**c** and **d**) during the growing seasons of 2013 and 2014.

The daily precipitation (P) and PET in the study period from the 1^st^ May to the 30^th^ September in 2013 and 2014 are shown in Fig. 1. The precipitation in the growing season of 2013 was high, with a total of 718.0 mm. The precipitation in the growing season of 2014 was relative low, with a total of 484.0 mm, and unevenly distributed with just 29 mm in May, 80 mm in June, 78 mm in July, 116 mm in August and 181 mm in September. The total PET in the study period was 332.18 mm in 2013 and 348.1 mm in 2014, respectively, with a daily mean PET of 2.17 mm·day^−1^ in 2013 and 1.80 mm·day^−1^ in 2014. The daily PET fluctuated strongly, e.g., within the range of 0.13–4.89 mm·day^−1^ in 2014.

**Figure 2:**
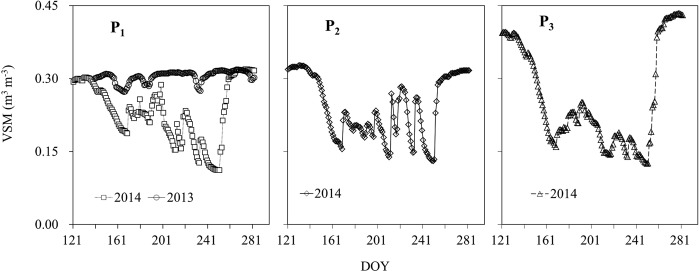
Variation of daily volumetric soil moisture of the 0–100 cm layer in the growing season of 2013 and 2014.

Figure 2 shows the seasonal variation pattern of VSM in the 0–100 cm soil layer during the study period. There was a common tendency of three-stage variation of “rapid consumption - continued volatility - recovery” for all plots in 2014. However, a significant difference in soil moisture quantity existed among plots, e. g., the highest values of VSM at the beginning and end of the study period in 2014 were significantly larger in P_3_ than in P_2_ and P_1_, because of the relatively higher soil capillary porosity in P_3_. The VSM fluctuated strongly from DOY 165 to 248 as the result of counteracting rainfall input and evapotranspiration output. In the period after DOY 248, the VSM was lifted rapidly and then kept at a high level, due to sufficient recharge from heavier rainfall and reduced evapotranspiration. This suggested that the VSM differences among plots and with time were influenced by weather, vegetation growth, evapotranspiration and the physical features of soil. It should be noted that the VSM in P1 in 2013 was obviously higher and more stable than that in 2014 due to the high and evenly distributed precipitation in 2013.

**Figure 3:**
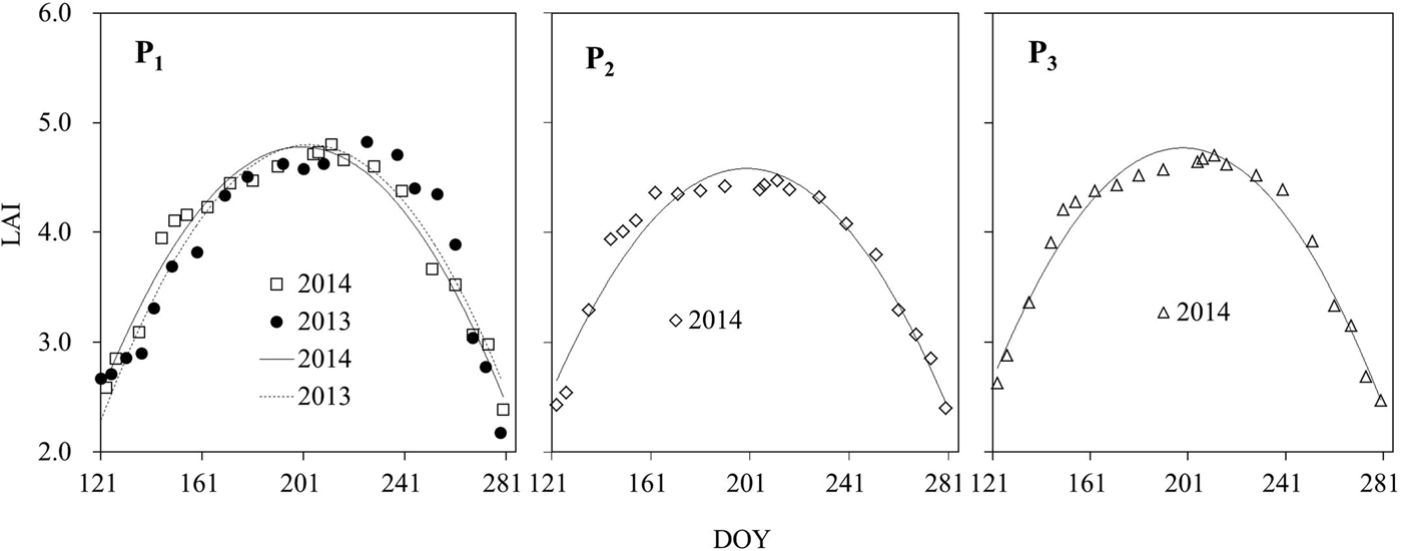
Variation pattern of forest canopy LAI in the growing seasons of 2013 and 2014.

Canopy LAI is an important vegetation factor influencing forest transpiration. The seasonal variation pattern of LAI of all plots presented the same trend of “increase – decrease” (Fig. 3), but there was some difference in the peak values and variation processes of LAI among plots due to their difference in site environment. For example, the peak LAI was 4.80, 4.47, 4.70 and the minimum LAI was 2.39, 2.40, and 2.47 in P_1_, P_2_ and P_3_ during the growing season of 2014.

### Variation of transpiration

**Figure 4:**
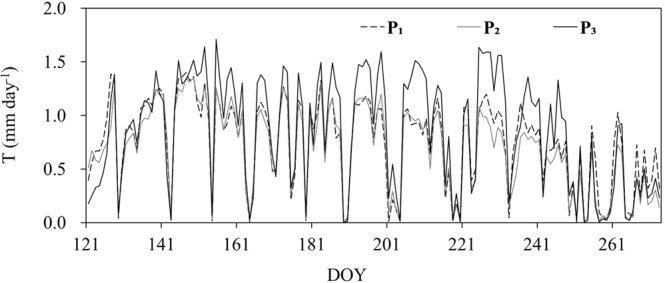
Seasonal variation pattern of daily transpiration of larch plantation in three plots in 2014.

The seasonal variation trend of daily T was similar among the 3 plots in the growing season of 2014 (Fig. 4), but the difference in their quantities was significant (paired t-test, P < 0.01). The daily T showed firstly a drastic increase prior to the DOY of 150, then a relatively stable period during the DOY of 151–235, and thereafter a decrease until the end of the growing season. The total T for the study period (DOY 121–273) was 114.28 mm in P_3_, 101.21 mm in P_1_, and 96.58 mm in P_2_. The peak daily T during the study period for P_1_, P_2_ and P_3_ was 1.40 (DOY 147), 1.35 (DOY 149) and 1.71 mm·day^−1^ (DOY 155), respectively, and the corresponding daily averages were 0.75, 0.69 and 0.86 mm·day^−1^. The T difference among plots was likely caused by the comprehensive influence of different soil moisture and canopy LAI.

### Response of T to PET

**Figure 5:**
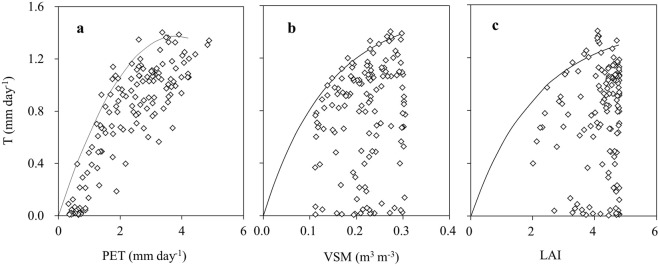
Response of daily T in P_1_ to the variation of PET, VSM of 0–100 cm soil layer and canopy LAI (solid line as upper boundary line).

Figure 5a shows the response of daily T to the variation of daily PET, with P_1_ as an example. The fluctuation range of T increases with rising PET. When using linear regression with an intercept forced to be zero, the T is highly related with PET (R^2^ = 0.70; slope = 0.321), indicating PET can explain 70% of the T variation. However, only the upper boundary line^35^ can present the real T-PET relation, by ignoring the interferences of soil moisture (VSM) and canopy transpiration capacity (LAI). Based on the selected upper boundary data of T which are at least one standard deviation larger than the mean T within each PET interval (0–1, 1–2, …, 4–5 mm·day^−1^), a regression analysis yielded a highly significant quadratic relation as below, which can explain a high proportion of the T variance (n = 10, R^2^ = 0.965):

1 $$T=-\,0.095\times PE{T}^{2}+0.723\times PET$$

According to the upper boundary line, the T firstly increases quickly at an almost linear rate with rising PET until 2.65 mm·day^−1^, thereafter the increasing rate decreases gradually within the PET range of 2.65–3.80 mm·day^−1^, and finally the T begins to approach a constant maximum with further PET increase. This indicates that the PET acts as a primary driver of T only when the PET is below its threshold of 3.80 mm·day^−1^.

### Response of T to VSM

The daily T and VSM in P_1_ are plotted in Fig. 5b which shows that the daily T was highly scattered for any given soil moisture, due to strong influence from other factors. However, the upper boundary line (solid line in Fig. 5b) indicates a clear and non-linear increase of T with rising VSM. This can be well described by the saturated exponential equation (Eq. (2), n = 13, R^2^ = 0.987). Within the VSM range of 0.08–0.22 m^3^·m^−3^, the boundary line increases near linearly with rising VSM at the rate of 0.12 mm·day^−1^ per 0.02 of VSM; whereas in the VSM range of 0.22–0.28 m^3^·m^−3^, T increases slowly within a small range of 1.33–1.37 mm·day^−1^, and thereafter the boundary line remains relatively unchanged. This indicates that there is a VSM threshold of 0.28 m^3^·m^−3^, below which the T will be limited by the soil water shortage.

2 $$T=1.504\,\ast \,(1-{e}^{-8.116\times VSM})$$

### Response of T to LAI

The daily T and LAI data in P_1_ are plotted in Fig. 5c which shows that the T displayed a high degree of scatter for any given LAI, due to the strong influence of other factors. To show the influence of varying LAI on T more clearly, the upper boundary line was used to analyze the T-LAI relation, and a significant saturated exponential equation was fitted (Eq. (3), n = 13, R^2^ = 0.979):

3 $$T=1.453\,\ast \,(1-{e}^{-0.466\times LAI})$$

When LAI varies within the range of 2.0–3.7, the boundary line increases nearly linearly with rising LAI; thereafter the T increases gradually and finally tends to its potential maximum. This indicates that there is a LAI threshold of 3.70, above which LAI is no longer the dominant driver for T variation, but other factors are of paramount importance, such as VSM and PET.

### A model estimating the daily T

In this study it was assumed that all the factors influencing forest T can be attributed to three aspects: the evapotranspiration pull from air (PET), the water supply ability from root zone soil (0–100 cm VSM), and the water conveyance capacity of trees (canopy LAI). To construct an integrated model which can describe the T response to PET, VSM and LAI, we coupled Eqs (1), (2) and (3) to form a general model (see Eq. (10) in Materials and methods). However, the values of all model parameters were newly fitted using field data in P_1_ during the entire observation period in 2014, based on the parameter values in the relations of T to single factors as the initial value of the fitting process. This newly fitted general T model is expressed as Eq. (4):

4 $$T=(0.793PET-0.078PE{T}^{2})\times (1-{e}^{-0.272LAI})\times (1-{e}^{-9.965VSM})\,{{\rm{R}}}^{2}=0.91$$

**Figure 6:**
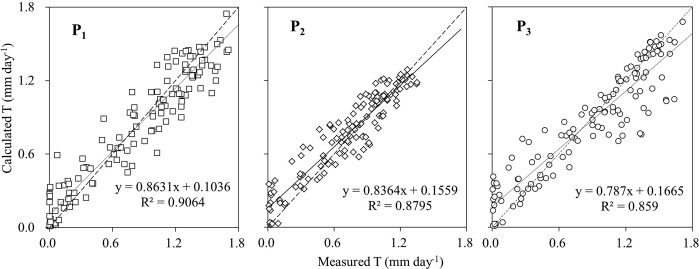
Comparison between model calculated and measured daily T in P_1_ (2013), P_2_ and P_3_ (2014). Dotted line is the 1:1 relation, solid line is the relation between calculated and measured values.

The sum of daily T calculated using Eq. (4) was 108.95 mm in P_1_ from DOY 140–273 of 2013, only 1.20 mm lower than the measured T (110.15 mm) during the study period. The ratio between the calculated and measured value is 93.46% in May, 100.34% in June, 108.99% in July, 93.52% in August, and 99.86% in September. The daily residuals between measured and calculated T varies mainly between −0.51 and 0.44 mm·day^−1^ (Fig. 6), with a slight overestimation which increases with lowering daily T. The coefficient of Nash and Sutcliffe was 0.87. However, the model would overestimate the daily T when the measured T fell below 0.35 mm·day^−1^.

To further validate the effectiveness of Eq. (4), the calculated daily T values were compared with measured data in P_2_ and P_3_ from DOY 121–273 of 2014, and they agree well (R^2^ = 0.88 in P_2_, 0.86 in P_3_) (Fig. 6). The sum of calculated T in P_2_ and P_3_ was 102.98 and 118.97 mm, which is only 6.22% higher and 2.11% lower than the measured values, with a satisfactory coefficient of Nash and Sutcliffe of 0.83 and 0.82, respectively.

From the above analysis it can be said that the model has the ability to accurately predict the daily T which matches the measured data well (R^2^ = 0.86–0.91 for 3 plots, Fig. 6), and covers a broad variation range of PET, VSM and LAI. So the model can well reflect and explain the influence of the main factors affecting the forest daily T.

### The impact of PET, VSM and LAI on T

From the observed weather data in 2010–2014 (data from 2010–2012 are not shown here), the maximum daily PET was picked out as the highest daily evaporative demand at the research site, with a value of 5.39 mm·day^−1^. In addition, we assumed the maximum VSM of 0.45 m^3^·m^−3^ and LAI of 5.00 based on previous studies at the same site. These maximum values of PET, VSM and LAI were then used to calculate the daily potential maximum T (T_max_) using Eq. (4). The sum of these calculated daily potential T in the whole study period is 226.44 mm, which reflects the potential total T when PET, VSM and LAI are not limited. These values will be used as a reference to assess the impact of each factor on forest T.

To assess the impact of individual factors on T, the actual data of the factor to be assessed in the study period in 2014, and the maximum values of other two factors were brought into Eq. (4) to calculate the daily T and their totals (T_t_) for P_1_, P_2_ and P_3_. Differences between the daily potential maximum T (T_max_) and their totals were calculated and used for this assessment (Table 1). The results show that the T reduction due to the limit of actual PET amounts to 80.78 mm (35.67%) in the study period for all plots; the corresponding T reduction is 26.30 (11.61%), 23.81 (10.51%) and 25.01 mm (11.04%) due to the insufficient VSM, and 25.71 (11.35%), 20.20 (8.92%) and 23.71 mm (10.49%) due to the insufficient LAI in P_1_, P_2_ and P_3_, respectively. This indicates the dominant limiting factor is PET, followed by the VSM and LAI.

**Table 1:** The total T reductions (mm) in the study period of 2014 by the limit of single factors (PET, VSM, LAI) for P_1_, P_2_ and P_3_ compared with the total potential maximum T of 226.44 mm.

Plots No.	T calculated as VSM = 0.45, LAI = 5.00, and actual PET	T calculated as PET = 5.39, LAI = 5.00, and actual VSM	T calculated as PET = 5.39, VSM = 0.45, and actual LAI	T Reduction by the actual PET limit	T Reduction by the actual VSM limit	T Reduction by the actual LAI limit
P_1_	145.66	200.14	200.73	80.78 (35.67%)	26.30 (11.61%)	25.71 (11.35%)
P_2_		202.63	206.24		23.81 (10.51%)	20.20 (8.92%)
P_3_		201.43	202.73		25.01 (11.04%)	23.71 (10.49%)

## Discussion

### The simplification of T response to changing environmental conditions

In this study, the numerous factors influencing forest T were simplified and divided into three aspects: the PET reflecting the atmospheric evaporative demand, the VSM reflecting the water supply ability, and the canopy LAI reflecting the stand transpiration capacity. For a given stand with fixed site condition, these three aspects must be the most important driving forces for forest T. As a result of their joint influences, the daily T of the larch plantation located in a semi-humid region in this study varied in a wide range of 0.01–1.71 mm·day^−1^ during the growing season of 2014. However, the response of T to individual factors (PET, VSM and LAI) is hard to derive and quantify when using a simple response-surface analysis of field data and the determination of corresponding thresholds of these factors is even not possible^36^. Therefore, the technique of upper boundary line was applied in this study.

Meteorological factors are the major drivers of T, as shown in Table 3 based on this study. Many relevant studies have involved numerous meteorological factors, including temperature, solar radiation, VPD, etc^25,28,29,37,38^. However, the research results have differed greatly, due to the large spatio-temporal variation of dominant meteorological factors. Using PET as an integrated indicator of the atmospheric evaporative demand, the comprehensive impact on T of multiple meteorological factors can be easily expressed and it is better than using any single meteorological factor^13,32,38^.

### The T response to single factors and related thresholds

The effect of PET on daily T of a larch plantation was analyzed in this study using the upper boundary line technique and it was found to follow a quadratic equation. The daily T increases firstly almost linearly with rising PET until its threshold of 3.80 mm·day^−1^, thereafter the T does not increase appreciably but gradually towards its potential maximum. Such a tendency of T response to PET in our study is consistent with other studies^15,33^. The PET threshold of 3.80 mm·day^−1^ in our study is slightly lower than that (4.00 mm·day^−1^) reported by Li *et al*.^33^ for a 28-year-old larch plantation at the semi-arid area of Liupan Mountains, probably due to the wetter climate in our semi-humid study site. Wu *et al*.^25^ found that the variation of daily T of black locust seedlings with PET follows a logistic function, and the PET threshold varies with soil texture, e.g., 3.2 and 4.0 mm·day^−1^ for a loamy clay soil and sandy loam soil, respectively under field conditions; whereas 3.6 and 3.9 mm·day^−1^ for a sandy loam soil and loamy clay soil, respectively within climate-controlled chambers. However, some studies have found that the T increases nearly linearly with rising PET, e.g., the reported average T/PET ratio of 0.55 for a *Pinus pinaster* forest when PET varied within 0–7 mm·day^−1^ in southern France^14^, and of 0.75 for a natural rain forest^39^. This is likely due to the high humidity in the study area and the small stomata inhibition of VPD.

Soil moisture determines the quantity of soil water available for transpiration, and its influence on T observed here is similar to the findings of Oren and Pataki^22^ in a deciduous forest, of Bindi *et al*.^24^ in 4-year-old grapevines in pots, and of Li *et al*.^33^ in a larch plantation. The daily T increases with rising soil moisture (expressed as VSM or REW) following non-linear models (like logistic function) or segmented linear models, firstly increasing very rapidly until a threshold of soil moisture is reached, and then leveling off to its maximum. However, the values of VSM threshold differ among these studies. For example, 0.15 m^3^·m^−3^ in the study of Ungar *et al*.^15^ for an Aleppo pine (*Pinus halepensis* Mill.) stand in Israel, a REW of 0.40 in a sugar maple (*Acer saccharum* Marsh.) forest near Quebec City in Canada^23^, a REW of 0.40 in a shrub (*Caragana korshinskii*) on the Loess Plateau of China^30^, 0.19 m^3^·m^−3^ (equal to 0.45 in REW) in a 28-year-old larch plantation in the semiarid area of Liupan Mountains^33^. In our study, the VSM threshold was determined as 0.28 m^3^·m^−3^ (equal to 0.51 in REW) which is higher than all the threshold values mentioned above. The causes for this difference may be the difference in the T-VSM response and its influencing factors, such as tree species^22,28^, soil texture^23,25^ and meteorological condition^25,32,33^. According to a potted corn study^40^, the value of VSM threshold increased from 0.23 to 0.34 m^3^·m^−3^ when the potential transpiration rate increased from 1.4 to 6.4 mm·day^−1^; Wu *et al*.^25^, based on a chamber seedling experiment, reported that the VSM threshold varied in the range of 0.213–0.222 m^3^·m^−3^ for loamy clay soil and of 0.125–0.145 m^3^·m^−3^ for sandy loam soil at different PET levels.

The forest canopy LAI, which varied greatly during the growing season, can directly affect the forest T. Several studies have reported the response of daily T to LAI. Bucci *et al*.^18^ found that the response of stand T to LAI (0 < LAI < 3.0) was well described by a saturated exponential equation at 5 Brazilian savanna (Cerrado) sites, with a LAI threshold of about 2.5 above which the T does not obviously increase. Forrester *et al*.^38^ reported that the stand T increased with rising LAI in the range of 1.0–6.0 in young *Eucalyptus nitens* plantation at ages of 5.1 and 6.3 years in a humid area. However, a contrasting result was reported by Xiong *et al*.^20^ studying a 24-years-old larch plantation in the semi-humid area of Liupan Mountains, possibly due to the high compensation from light and soil moisture. Compared with that observed by Bucci *et al*.^18^, our study found a higher LAI threshold of 3.8, but the same tendency of T response to LAI. The lower LAI threshold in the study of Bucci *et al*.^18^ can be partly caused by the limitation of soil nutrient availability. However, the increasing competition for light and soil water^20^ or limiting meteorological factors^33^ could be the dominant causes of the declining response rate of T with rising LAI in our study. The effect of canopy structure and physiology on T can be predominantly caused by the factors of LAI^18^, stomatal conductance^39^ and effective transpiration capacity of leaves. However, only the effect of varying LAI was considered in our study, while the other two factors were excluded. These may be the dominant causes of the over- or under-estimation of the daily T by our model in the current study.

The above discussion indicates that the tendency of T response to rising PET, VSM and LAI among the different study areas and vegetation types is mostly consistent. However, the threshold values of these factors vary with plant type, tree species, vegetation condition, soil condition, climate type and so on. In addition, some of the existing studies were conducted in chambers (e.g., Wu *et al*.^25^) or pots (e.g., Denmead & Shaw^40^) usually under controlled weather conditions. In the limited field studies, the data from cloudy, overcast, raining days or those with extreme VPD were excluded^16,26,27^, the canopy variation was not considered (e.g., Li *et al*.^33^), or the plots were treated with silvicultural interventions (e.g., Forrester *et al*.^38^). Therefore, these study results will be surely far different from the T behavior of trees in the field under natural conditions. Hence many further studies are required in future to look for the universal T response to the changing environmental and vegetation conditions.

### Daily forest T model

Although many different field observation methods for getting stand T were used^37,39,41^, the complete and accurate field data are still limited. To overcome this, mechanism-based T models should be developed and applied for more precise prediction^13,18,28,33^.

Many studies have tried to develop a stand T model based on field observations^22,27,28,31,39,42,43^. However, they have mostly considered just one or two of the main influencing factors (soil moisture, meteorological factors, vegetation condition, etc.), and the model structure was simple with the focus on reproducing T for a specific site. Such simplified models without coupling all the main influencing factors (PET, VSM, LAI), as shown in this study, cannot be widely applied under changing environmental conditions.

The T model developed in this study can predict the daily T of forests with an accuracy higher than or close to most of the models established previously in similar studies^22,28,39,42,44^. For example, the models including LAI, VPD within canopy, photosynthetically active radiation above canopy and soil moisture depletion, explained 75% and 81% of the T variation in the studies of Oren & Pataki^22^ and Phillips & Oren^44^. In the short period study during the middle of the growing season, when the LAI was relatively stable and the influence of some abiotic environmental factors was low^33^, the model describing the daily T response to the PET and REW for a larch plantation could explain 91% of the T variation.

The mechanism-based T model developed in this study has great potential for estimating the daily T of larch plantations under widely varying site conditions (slope position, slope aspect, etc.) if data on the main influencing factors (PET, VSM and LAI) are available. This model can also promote the deeper understanding of forest-water interactions and integrated forest-water management. Moreover, the model application can be enlarged through coupling this model with other models describing the variation of PET, VSM and LAI. Finally, the concept and framework of this model can be taken as a reference for developing similar models for other tree species and vegetation types in different regions.

The model established in this study can be further improved by the inclusion of other factors in the model framework, such as the mountain terrain shade, the depth distribution of tree roots^45^ and soil water^13^, plant diseases and insect pests^12^, the water deficit stress^24^, and the leaf injury from low temperatures. Furthermore, we do not have enough T data for larch plantations in different regions with varying forest structure, tree age, climate, soil texture and hydrological features, for fitting the model parameters and testing the broad applicability of this model. Therefore, more studies are required for improving and refining this model and its application.

## Conclusions

The daily transpiration (T) of a larch plantation at one semi-humid site of northwest China showed a big variation, within the range of 0.01–1.71 mm·day^−1^ in the growing season. To understand and predict the daily T easily, the numerous influencing factors were simplified into three aspects: the potential evapotranspiration (PET) representing the atmospheric evaporative demand, the volumetric soil moisture (VSM) within the main root zone representing the soil water availability, and the forest canopy leaf area index (LAI) representing the stand transpiration capacity. Based on the analysis of upper boundary lines, the daily T increases with rising PET, VSM, and LAI firstly rapidly, then gradually and tends to be stable when a threshold is reached. The function types of the daily T response were also determined, i.e., a quadratic equation for PET and a saturated exponential function for VSM and LAI. A mechanism-based daily T model was developed through coupling the T response functions to the widely varying PET, VSM and LAI, and the model parameters were fitted based on the field measured data. This model can not only well predict the daily T of forests, but also explain 86–91% of the highly scattered variation in the daily T of the 3 larch plantation plots. Although this study was carried out just at one site with limited plantation plots and over a short time period, the concept and framework of this model might be a reference for other studies in different regions.

## Materials and Methods

### Study site

The study site is located in the southern part of the Liupan Mountains (106°15′ E, 35°29′ N) in northwest China, which forms the important headwater area of several tributaries of the Yellow River. The study was carried out at the small watershed of Xiangshuihe (XSH), which has an area of 43.74 km^2^ and an elevation range of 1960–2860 m a.s.l. The predominant soil type is hapli-ustic argosols, with sandy loam texture, and the thickness range of 50–300 cm according to site condition. The climate here is semi-humid continental monsoon, with a dry-cool winter and a warm-wet summer. According to the records from 1960–2010 at Jingyuan Weather Station about 7 km away from the study sites, the mean annual air temperature was 6.0 °C and the mean annual precipitation (P) was 610 mm with 87% concentrated in the period from June to September.

Since the 1980s, the forest coverage in XSH has been greatly increased by afforestation, with the main purposes to produce more timber and protect soil against erosion^46^. The plantation of Prince Rupprecht’s larch, one dominant afforestation tree species in mountain areas of northwest and north China, is the dominant artificial forest, which accounts for 23.62% of the watershed area and is distributed mostly on shady, semi-shady and semi-sunny slopes.

### Plot setup

In this study, one representative plot of larch plantation was set up in 2014 at the upper slope (P_1_) and lower slope (P_2_), respectively, on a southeast-facing hillslope with a gradient of 36.4°; meanwhile, another plot (P_3_) was set up at the lower slope in 2013 on a south-facing hillslope with a gradient of 33.5°. The size of all plots was 30 m (width) × 30 m (slope length). An elevation difference of 117.6 m exists between P_1_ and P_2_. The plots P_2_ and P_3_ are at similar elevation, but separated by a horizontal distance of 210.6 m (Table 2). The micro-relief within plots is relatively uniform, without any obvious humps or concaves. The soil thickness is more than 100 cm for all plots. The mean bulk density of the 0–100 cm soil layer is around 1.1 g·cm^−3^.

**Table 2:** General site, vegetation and 0–100 cm soil layer descriptions of the larch plantation plots.

Plot No.	Elevation m	Slope position	Stand density trees·ha^−1^	Canopy density	Mean tree height m	Mean tree DBH cm	Canopy LAI	Soil bulk density g·cm^−3^	Total soil porosity %	Soil capillary porosity %	Field capacity %, v
P_1_	2394.2	Middle	930	0.74	19.07	20.49	2.39–4.80	1.21	48.91	32.70	36.65
P_2_	2276.6	Lower	933	0.75	16.00	18.85	2.40–4.47	1.11	51.73	37.91	45.50
P_3_	2283.1	Lower	1139	0.80	14.85	18.48	2.47–4.70	1.09	51.40	41.21	46.17

All the trees in each plot had the same age of 33-years in 2014. The canopy density of the 3 plots was similar, varying within 0.74–0.80. The understory shrubs were scattered due to the high canopy density. An herb layer was developed, with the coverage of about 40%. The tree density (stocking) in the plots varied within 930–1139 trees·ha^−1^, the mean diameter at breast height (DBH) and mean height of trees varied within 18.48–20.49 cm and 14.85–19.07 m, respectively. The canopy LAI was measured at 11 random points dispersed within each plot using a LAI-2000 (Li-Cor Biosciences, Lincoln, NE, USA) once per week from May to September in 2014. The LAI range was similar among the three plots at 2.39–4.80 for P_1_, 2.40–4.47 for P_2_, and 2.47–4.70 for P_3_, respectively.

### Sap flow measurement and sapwood area

**Figure 7:**
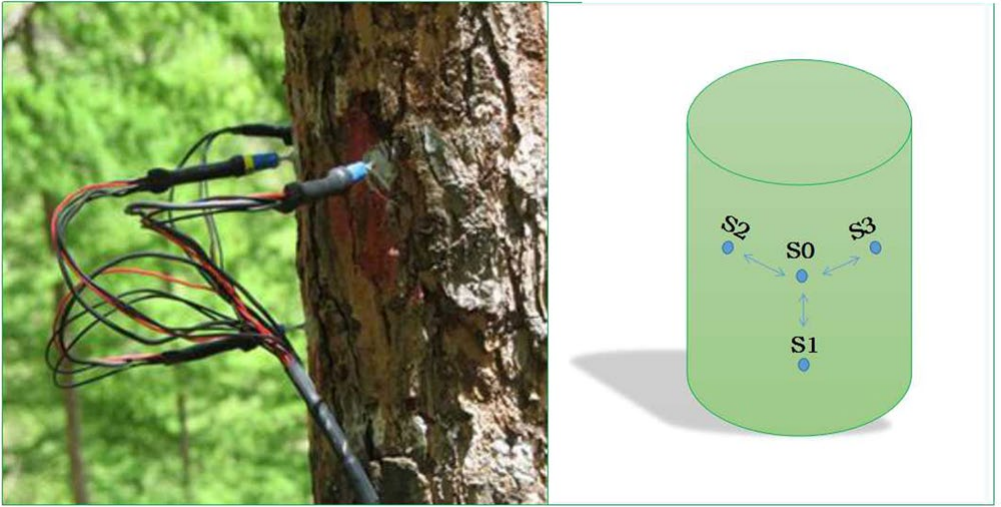
Radially inserted probes in hydro-active xylem of larch trunk to measure sap flow density.

Sap flow was measured at 8 healthy sample trees within different DBH classes in each plot, using thermal dissipation probes (SF-L, Ecomatik, Munich, Germany). Each set of probes consisted of 4 sensors (S1, a heated sensor, is powered by a constant current in 12 v voltage. S0, S2, S3 are reference sensors) with a length of 20 mm and a diameter of 2 mm (Fig. 7). They were installed at breast height (1.3 m above ground) on the northern side of each tree trunk; then covered with aluminum foil to prevent direct solar radiation and physical damage, and to minimize temperature fluctuation in the sapwood area. Sap flow data were recorded on a data logger (DL2; Delta-T Devices, UK) with a multiplexer every 30 s and averaged in every 5 min interval. For trees with a sapwood thickness over 20 mm, an additional system was installed at the sapwood depth of 20–40 mm from the cambium. The measurements were conducted in the entire growing season from May to September in 2014 (for P_1_, P_2_, P_3_) and 2013 (for P_1_ only).

**Figure 8:**
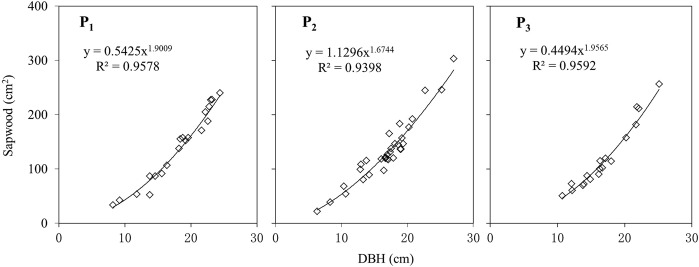
Variation of sapwood area with the DBH of individual trees in different plots.

The sapwood thickness (mm) of more than 20 trees in each plot was measured by the Lintab5 rings analyzer (Rinntech, Heidelberg, Germany) and its software TSAP (time series analysis presentations) based on the cores extracted with a 5-mm increment borer at breast height, and assessed as the mean of two orthogonal measurements. The boundary between sapwood and heartwood was identified by wood color difference. The empirical power function between sapwood area (A_S_, cm^2^) and DBH (cm) of trees was established for each plot (Fig. 8).

The sapwood-DBH equations in Fig. 8 and the DBH of all trees in each plot were used to calculate the A_s_. The sapwood area per hectare (m^2^·ha^–1^) was calculated as the sum of sapwood area of all trees in each plot, which was 16.30 in P_3_, 15.50 in P_1_, and 14.80 in P_2_ in 2014 (Table 3).

**Table 3:** Sapwood area of individual trees (A_S-tree_) and of stand (A_S-stand_) in 2014 at different plots.

Plot No.	Max A_S-tree_ cm^2^	Min A_S-tree_ cm^2^	Average A_S-tree_ cm^2^	A_S-stand_ m^2^·ha^−1^
P_1_	387.05	38.78	166.77	15.50
P_2_	321.96	49.85	150.13	14.80
P_3_	287.57	23.40	143.09	16.30

### Sap flow density and transpiration calculation

The sap flow density (J_s_) was calculated by Eqs (5) and (6) as described by Xiong *et al*.^20^. The raw data were transferred to J_s_ with the Baseliner Program (Version 3.0.7, C-H_2_O Ecology Lab, Duke University, Durham, NC, USA).

5 $${{\rm{J}}}_{{\rm{s}}}={\rm{0.714}}\times {(\frac{{{\rm{d}}}_{{\rm{tmax}}}}{{{\rm{d}}}_{{\rm{tact}}}}-{\rm{1}})}^{{\rm{1.231}}}$$

6 $${{\rm{d}}}_{{\rm{tact}}}={{\rm{d}}}_{{\rm{t0}}}-\frac{({{\rm{d}}}_{{\rm{t2}}}+{{\rm{d}}}_{{\rm{t3}}})}{{\rm{2}}}$$

where, d_tact_ is the difference in instantaneous temperature (°C) of the heated sensor and the reference sensors; d_tmax_ is the value of d_tact_ when sap flow is nil or close to zero; d_t0_ is the temperature difference between heated needle (S_1_) and unheated needle (S_0_) (°C), d_t2_ and d_t3_ are the temperature difference between S_1_ and the probes of reference pair (S_2_ and S_3_), respectively.

J_s_ was corrected according to Clearwater *et al*.^47^ when the tree sapwood thickness was less than the probe length. For the trees with a sapwood thickness over 20 mm, a radial profile of J_s_ was calculated based on the method described by Xiong *et al*.^20^.

The daily T (mm·day^−1^) was scaled up from sample trees to the stand level by Eq. (7).

7 $${\rm{{\rm T}}}={J}_{C}\times \frac{\sum {{\rm{{\rm A}}}}_{{\rm{s}}}}{{\rm{S}}\times 1000}\times 60\times 24$$

where, J_c_ (ml·cm^−2^·min^−1^) is the stand daily mean sap flux density which was computed as the sapwood area weighted average of J_s_ for each DBH class; S (m^2^) is the projected area of the plot; and ∑A_s_ (cm^2^) is the cumulative sapwood area of all trees within the plot.

### Weather and soil moisture measurement

An automatic weather station (Weatherhawk, USA) was placed in an open grassland, 110.9 m and 121.4 m away from the plot P_2_ and P_3_, respectively, to collect weather data including precipitation (P, mm), air temperature (T, °C), relative air humidity (RH, %), solar radiation (R, w·m^−2^) and wind speed (U, m·s^−1^). This weather station was 50 m beyond the forest edge, to minimize the canopy interference. The weather data were recorded every 5 min. The daily potential evapotranspiration (PET, mm) was calculated using the FAO Penman-Monteith calculation method^48^ for a reference grass surface, and directly obtained from the weather station.

Soil water potential (ψ, MPa) was monitored with the equilibrium tensiometer (EQ. 15; Ecomatik, Munich, Germany) positioned at the soil depths of 5, 15, 30, 50, 70 and 90 cm in the forest stand. Data were recorded every 5 min by a data logger (DL6; Delta-T Devices, UK) installed adjacent to the soil profile. The VSM (m^3^·m^−3^) was calculated from the raw data of ψ, by using their relation for each depth determined by the method of centrifugation with soil cylinder cores collected in the same study site^49^. The weighted average of VSM in the 0–100 cm soil layer was calculated based on the measured VSM in different soil depths using Eq. (8):

8 $${{\rm{VSM}}}_{0\mbox{--}100}=({{\rm{VSM}}}_{5}+{{\rm{VSM}}}_{15}+2{{\rm{VSM}}}_{30}+2{{\rm{VSM}}}_{50}+2{{\rm{VSM}}}_{70}+2{{\rm{VSM}}}_{90})/10$$

In addition, in order to apply the results to other regions with different soil hydrological properties, and also to ensure the results are comparable with other studies, VSM was converted into the REW using Eq. (9) as described by Granier^50^:

9 $${\rm{REW}}=({\rm{VSM}}-{{\rm{VSM}}}_{{\rm{m}}})/({{\rm{VSM}}}_{{\rm{FC}}}-{{\rm{VSM}}}_{{\rm{m}}})$$

where, VSM is the actual volumetric soil moisture in the root zone, VSM_m_ is the value of VSM at the wilting point (ψ = −1.5 MPa), and VSM_FC_ is the VSM at the field capacity (ψ = −0.01 MPa).

### An integrated model for daily T estimation

The model framework was adapted from the functions expressing the response of stomatal conductance to environmental variables^51^ and later used for T estimation in the studies of Oren & Pataki^22^ and Phillips & Oren^44^, using Eq. (10):

10 $${\rm{T}}={f}_{1}({\rm{PET}})\cdot {f}_{2}({\rm{VSM}})\cdot {f}_{3}(\mathrm{LAI})$$

where, *f*_1_, *f*_2_, *f*_3_ are functions describing the variation of T with PET, VSM and LAI. Firstly, the concrete types of these functions were determined separately using the upper boundary line analysis of raw data observed in the field (Ram Oren’s H_2_O Ecology Group in Duke University). A detailed description of this analysis can be found in Xiong *et al*.^20^. Then, the values of the parameters in Eq. (10) were fitted based on field data at P_1_ in 2014, and the model was validated using the field data at P_1_ in 2013 and at P_2_ and P_3_ in 2014. The fitting quality of the model was evaluated by a non-dimensional efficiency criterion of Nash and Sutcliffe^52^.

### Statistical analysis

The SPSS software package (version 19.0 for Windows, SPSS Inc., USA) was used to determine the variance of T and VSM among different plots, and to realize the best-fit of the T model (Eq. (10)) which links the T variation to the PET, VSM and LAI.

## Data Availability

The datasets generated during and/or analyzed during the current study are available from the corresponding author on reasonable request.
